# Chiari 1 Malformation Presenting as Central Sleep Apnea during Pregnancy: A Case Report, Treatment Considerations, and Review of the Literature

**DOI:** 10.3389/fneur.2014.00195

**Published:** 2014-10-24

**Authors:** Erik K. St. Louis, Praveen Jinnur, Stuart J. McCarter, Ethan J. Duwell, Eduardo E. Benarroch, Kejal Kantarci, Mark A. Pichelmann, Michael H. Silber, Bradley F. Boeve, Eric J. Olson, Timothy I. Morgenthaler, Virend K. Somers

**Affiliations:** ^1^Mayo Center for Sleep Medicine, Division of Pulmonary and Critical Care Medicine, Mayo Clinic, Rochester, MN, USA; ^2^Section of Sleep Neurology, Mayo Clinic, Rochester, MN, USA; ^3^Department of Neurology, Mayo Clinic, Rochester, MN, USA; ^4^Department of Medicine, Mayo Clinic, Rochester, MN, USA; ^5^Sleep Clinical Research Unit Laboratory, Mayo Clinic Hospital, Mayo Clinic, Rochester, MN, USA; ^6^Department of Radiology, Mayo Clinic, Rochester, MN, USA; ^7^Department of Neurosurgery, Mayo Clinic, Rochester, MN, USA

**Keywords:** Chiari malformation, central sleep apnea, pregnancy, presentation, adaptive servoventilation

## Abstract

**Purpose:** Chiari malformation (CM) type-1 frequently causes obstructive or central sleep-disordered breathing (SDB) in both adults and children, although SDB is relatively rare as a presenting manifestation in the absence of other neurological symptoms. The definitive treatment of symptomatic CM is surgical decompression. We report a case that is, to our knowledge, a novel manifestation of central sleep apnea (CSA) due to CM type-1 with severe exacerbation and initial clinical presentation during pregnancy.

**Methods:** Case report from tertiary care comprehensive sleep medicine center with literature review of SDB manifestations associated with CM type-1. PubMed search was conducted between January 1982 and October 2013.

**Results:** We report a 25-year-old woman with severe CSA initially presenting during her first pregnancy that eventually proved to be caused by CM type-1. The patient was successfully treated preoperatively by adaptive servoventilation (ASV), with effective resolution of SDB following surgical decompression, and without recurrence in a subsequent pregnancy. Our literature review found that 58% of CM patients with SDB had OSA alone, 28% had CSA alone, 8 (10%) had mixed OSA/CSA, and 6 (8%) had hypoventilation. Of CM patients presenting with SDB, 50% had OSA, 42% had CSA, 8% had mixed OSA/CSA, and 10.4% had hypoventilation. We speculate that CSA may develop in CM patients in whom brainstem compression results in excessive central chemoreflex sensitivity with consequent hypocapnic CSA.

**Conclusion:** Chiari malformation type-1 may present with a diversity of SDB manifestations, and timely recognition and surgical referral are necessary to prevent further neurological deficits. ASV therapy can effectively manage CSA caused by CM type-1, which may initially present during pregnancy.

## Introduction

Chiari malformation (CM) type-1 frequently causes obstructive or central sleep-disordered breathing (SDB) in both adults and children, although SDB is relatively rare as a presenting manifestation in the absence of other neurological symptoms. To our knowledge, central sleep apnea (CSA) due to CM type-1 with severe exacerbation and presentation during pregnancy has not been previously reported. The definitive treatment of symptomatic CM is surgical decompression. Here, we report a case with successful preoperative treatment by adaptive servoventilation (ASV), effective resolution of SDB following surgical decompression and with no recurrence in a subsequent pregnancy. We also discuss relevant pathophysiologic considerations and review the previous literature concerning the range of presenting SDB manifestations and surgical treatment outcomes associated with CM type-1.

## Background

**Figure 1:**
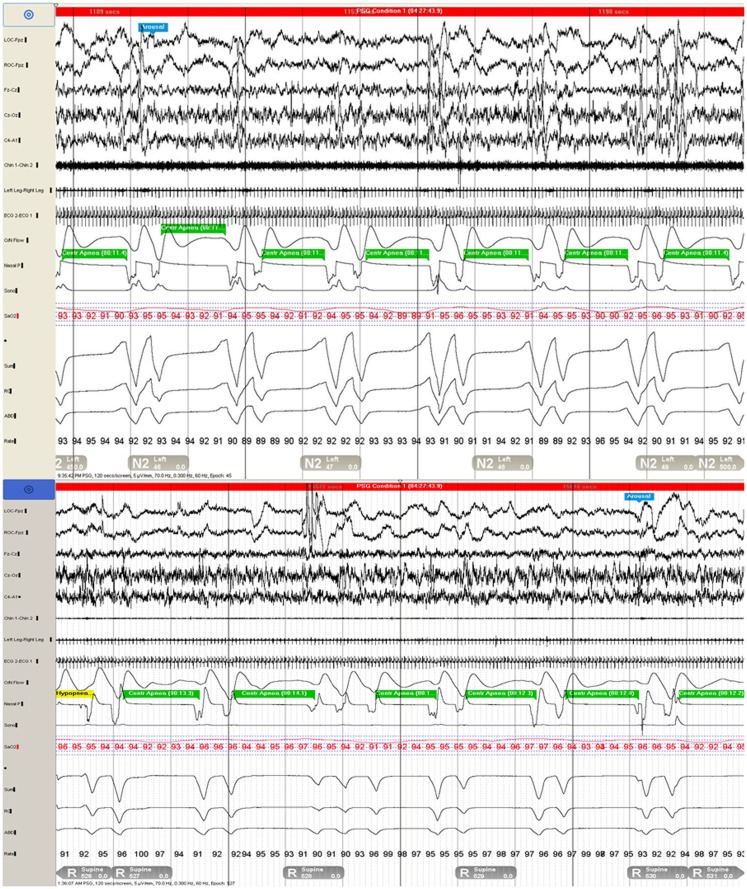
**Polysomnogram demonstrates repetitive central apneas during two 120-s epochs during N2 (top) and REM (bottom) sleep**.

**Figure 2:**
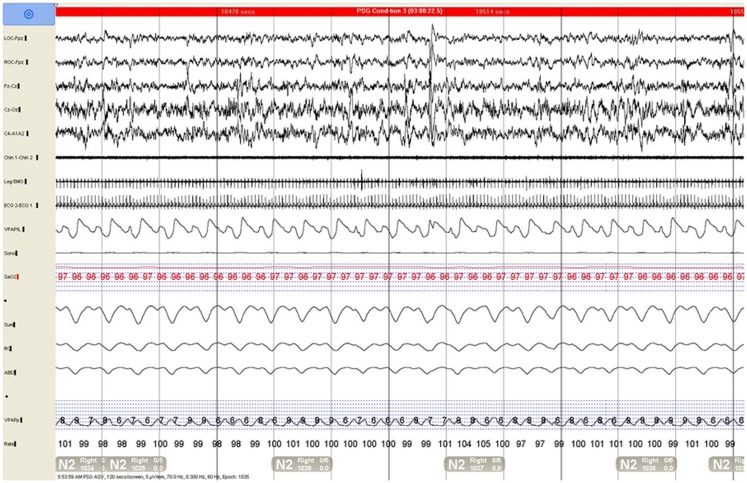
**Adaptive servoventilation (ASV) completely resolved central disordered breathing events and snoring and improved sleep consolidation**.

A 25-year-old primigravid woman at 22 weeks gestation presented for evaluation of witnessed pauses in breathing during sleep. She had been told of past snoring by her husband, which had increased as her pregnancy progressed. Her Epworth sleepiness scale (ESS) score was 10. Vital signs included a BMI of 30.4 kg/m^2^, blood pressure of 110/76 mmHg, and a respiratory rate of 12/min. Physical exam was positive for a Friedman grade III-palate position with a neck circumference of 35 cm. Polysomnography revealed an apnea–hypopnea index (AHI) of 155/hour, with central apnea index (CAI) of 142/h (Figure 1; Table 1), mean oxyhemoglobin saturation of 94%, and an arousal index (AI) of 75/h. Nasal continuous positive airway pressure (CPAP) improved oxygenation, yet, CAI increased to 155/h and AI increased to 115/h. ASV with end-expiratory pressure (EPAP) set at 6 cm H_2_O and automatically delivered inspiratory pressure support (IPAP) set to a range between 3 and 15 cm water, and respiratory rate set to the default automation completely resolved SDB events and snoring, improving sleep consolidation with AI being reduced to 18.6/h (Figure 2). She was treated with ASV therapy throughout the remainder of her pregnancy and gave birth to a healthy baby girl at term by normal spontaneous vaginal delivery. Three months post-partum, she noted improvement in sleep quality and daytime sleepiness with an ESS score of 7. Complete neurological examination and transthoracic echocardiogram were normal. Repeated polysomnography (Table 2) demonstrated an AHI of 12/h (all central apneas), and ASV again resolved all SDB. Quantitative analysis of REM muscle tone according to established methods (1) revealed elevated phasic muscle activity of 22.5% (whereas similarly aged female controls aged 25–32 years old showed average phasic activity of 10.2%, tonic activity in both our patient and controls was 0%), and automated submentalis REM atonia index (2) was also borderline at 0.91. However, there was no history of dream enactment behavior. She elected to continue therapy with ASV.

**Table 1:** **Polysomnogram 1 during pregnancy**.

	Diagnostic	Therapeutic (CPAP)	Therapeutic (ASV)
SE (%)	81.2%	36.4	64.5
TST (min)	217.5	24.0	116.4
N1 (%)	26	64.6	8.2
N2 (%)	54	35.4	49.0
N3 (%)	9.4	0	8.5
REM (%)	10.6	0	34.4
AI (events/h)	75.0	117.5	18.6
Total AHI (events/h)	155.0	155.0	0
Total CAI (events/h)	142.0	155.0	0
Supine AHI (events/h)	169.0	155.0	0
Non-supine AHI (events/h)	158.0	(Not sampled)	0
SaO_2_ mean (%)	94	97	97
SaO_2_ nadir (%)	86	92	94
SaO_2_ <90% (min)	7.8	0	0
PLMI (events/h)	0	0	0

*SE, sleep efficiency; TST, total sleep time; AI, arousal index; AHI, apnea–hypopnea index; CAI, central apnea index; NR, NREM, R, REM; SaO2, oxyhemoglobin saturation; PLMI, periodic limb movement index; PLMAI, periodic limb movement arousal index. All scoring above was performed in accordance with American Academy of Sleep Medicine 2007 rules (3)*.

**Table 2:** **Polysomnogram 2 during post-partum state**.

	Diagnostic	Therapeutic (ASV)
SE (%)	90.9%	92.2
TST (min)	155.0	270.9
N1 (%)	8.7	3.0
N2 (%)	54.2	38.4
N3 (%)	28.7	8.5
REM (%)	8.4	29.4
AI (events/h)	18.2	29.3
Total AHI (events/h)	12.0	0
Total CAI (events/h)	12.0	0
Supine AHI (events/h)	32.0	0
Non-supine AHI (events/h)	5.0	0
SaO_2_ mean (%)	95	96
SaO_2_ nadir (%)	90	92
SaO_2_ <90% (min)	0	0
PLMI (events/h)	0	0

*SE, sleep efficiency; TST, total sleep time; AI, arousal index; AHI, apnea–hypopnea index; CAI, central apnea index; NR, NREM, R, REM; SaO2, oxyhemoglobin saturation; PLMI, periodic limb movement index; PLMAI, periodic limb movement arousal index. All scoring above was performed in accordance with American Academy of Sleep Medicine 2007 rules (3)*.

**Figure 3:**
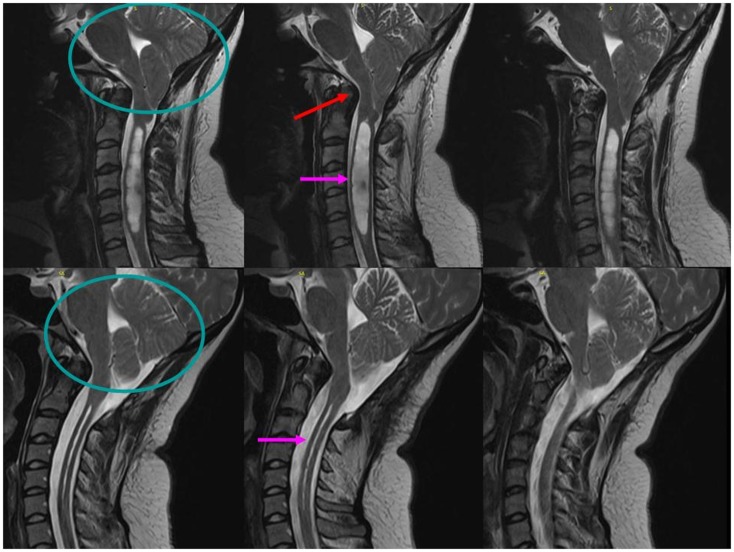
**Sagittal T2-weighted MRI scans of the cervical spine demonstrate significant posterior fossa volume reduction (circle) with basilar invagination (red arrow) and a Chiari 1 malformation with 2.2-cm cerebellar tonsillar protrusion below the foramen magnum (circle), resulting in pontomedullary compression and a 1.2-cm maximal diameter cervical cord syrinx (pink arrow, top)**. Following suboccipital craniectomy and C1–2 laminectomy with duraplasty surgery, the post-operative scans show an increased posterior fossa volume (circle) with adequate decompression of the brainstem and cervical syrinx to a maximal diameter of 12 mm (pink arrow, bottom).

The following year, she reported the new symptom of intermittent hiccups and, in retrospect, affirmed exertional or cough-related posterior–occipital–nuchal regional headaches. Evolution of these new symptoms prompted brain MRI, which revealed a CM type-1 with cerebellar tonsillar protrusion and cervical cord syrinx. Hydrocephalus was not observed (Figure 3). Subsequently, she underwent suboccipital craniectomy, C1–2 laminectomy, and duraplasty. At post-operative follow-up 3 months later, her symptoms of snoring and SDB were entirely resolved. Repeated MRI showed substantially increased volume in the posterior fossa, with improvement in the extent and maximal diameter of the cervical syrinx cavity (Figure 3). Repeated polysomnography (Table 3) demonstrated an AHI of 6/h (all postarousal/sleep-onset central apneas). Two years post-surgery, she returned during the first trimester of her second pregnancy. Follow-up polysomnography (Table 3) showed only primary snoring without significant residual SDB, with an AHI of 3/h. Quantitative REM phasic density was again 21.7% and RAI was borderline at 0.91.

**Table 3:** **Polysomnograms 3 and 4, post-operative study following suboccipital craniectomy and repeated study during pregnancy 2 (first trimester)**.

	PSG 3 (diagnostic)	PSG4 (diagnostic)
SE (%)	88.2%	88.8
TST (min)	336.0	409.0
N1 (%)	9.7	5.9
N2 (%)	43.0	51.5
N3 (%)	20.5	17.2
REM (%)	26.8	25.4
AI (events/h)	30.4	8.5
Total AHI (events/h)	6.0	3.0
Total CAI (events/h)	5.0	3.0
Supine AHI (events/h)	8.0	5.0
Non-supine AHI (events/h)	3.0	1.0
SaO_2_ mean (%)	95	96
SaO_2_ nadir (%)	92	92
SaO_2_ <90% (min)	0	0
PLMI (events/h)	3.7	0.6

*SE, sleep efficiency; TST, total sleep time; AI, arousal index; AHI, apnea–hypopnea index; CAI, central apnea index; NR, NREM, R, REM; SaO2, oxyhemoglobin saturation; PLMI, periodic limb movement index; PLMAI, periodic limb movement arousal index. All scoring above was performed in accordance with American Academy of Sleep Medicine 2007 rules (3)*.

## Discussion

Sleep-disordered breathing including obstructive sleep apnea (OSA) and CSA are common in CM, although other neurological symptoms and signs usually precede or accompany SDB abnormalities (4–8). Between 60 and 88% of CM type-1 patients have SDB, and patients with radiographic basilar invagination (BI) are particularly likely to manifest CSA (9). Isolated sleep apnea as a presenting manifestation associated with CM is rare and is usually more frequent in children than adults (4–8).

The severe, very high-frequency central SDB associated with CM in our case most likely resulted from direct compression of the brainstem, with serial improvement in the post-surgical polysomnograms consistent with improvement following decompressive surgery (2, 5–7, 9–15). Brainstem distortion probably caused a disturbance in the pontomedullary respiratory network function, particularly in the ventral medullary respiratory group, including the pre-Bötzinger complex, which is responsible for pacemaker activity and respiratory rhythm genesis. Respiratory abnormalities could be expected from disturbances of the dorsal medullary respiratory group at the level of the nucleus tractus solitarius, which integrates central afferents from peripheral chemo- and baroreceptors. Both areas are interconnected with the retrotrapezoid nucleus in the rostral ventral medulla, which is the primary central respiratory chemoreceptive area, and the pontine respiratory group, including the lateral parabrachial/Kolliker-Fuse complex, which is critical to shape the normal respiratory pattern of pre-inspiratory and expiratory phases of breathing via connections to different subpopulations of neurons of the ventral respiratory group (14). Neuroimaging demonstrated evidence for direct compression of the ventrolateral medulla (containing the ventral respiratory group) and distortion of the caudal dorsal medulla (including the area of the nucleus of the solitary tract) by BI and CM that was well decompressed by surgery.

Chiari malformation may result in REM sleep behavior disorder (RBD), presumably as another manifestation of brainstem dysfunction resulting from pontine compression. The presence of RBD was noted in 23 out of 103 patients with CM in a recent large series (2). While our patient never manifested clinically overt dream enactment behavior to suggest RBD, our quantitative analysis of chin muscle tone demonstrated increased quantitative REM sleep muscle tone in comparison to similarly aged women analyzed in our laboratory as well as to published cutoff values for defining abnormal degrees of phasic REM muscle tone (1). The increased REM sleep muscle tone in our patient likely resulted from CM-mediated brainstem compression and dysfunction of the nucleus reticularis gigantocellularis, which receives inputs from REM-on neurons of the neighboring sublaterodorsal, magnocellular reticular formation, laterodorsal tegmental, and pedunculopontine nuclei (which mediate REM atonia) (16). While decompressive surgery relieved radiographic medullary compression and cured CSA, abnormal levels of RSWA persisted after surgery.

The dramatic pregnancy-related worsening of CSA seen in our patient is striking and of uncertain cause. Progesterone surge during pregnancy may sensitize central respiratory centers, leading to respiratory alkalosis, and resultant hypocapnea (17). This provides a possible mechanism by which pre-existing CSA in a vulnerable patient such as ours could precipitously worsen in severity, leading to an overtly symptomatic presentation during pregnancy. Only a few reports of pregnancies with associated CM have been reported (18–20), and optimal anesthetic management and the mode of delivery (vaginal or cesarean) in parturient with syringomyelia and Arnold–Chiari malformation has not been established. General anesthetic risks in CM patients are difficulty with airway management, risk of hypoxia, and damage to the spinal cord by increased intracranial pressure caused by laryngoscopy and intubation. During epidural anesthesia in CM patients, risks include subarachnoid space compression caused by sudden distension of the epidural space or decompression by accidental dural puncture. In both cases, neurological damage may occur. It is thought that spinal anesthesia should be avoided in CM patients.

While hypocapnic CSA associated with congestive heart failure or neurological disorders is frequently relatively resistant to nasal CPAP therapy, ASV has been shown to be effective. A reduction in frequency of central apneic events, improvement in oxygenation, reduced sleep fragmentation, and reduced daytime sleepiness in compliant patients has been noted in retrospective case series (21, 22). CPAP was ineffective in our patient, but ASV resulted in complete control of central apneic events with improved oxyhemoglobin desaturation and sleep depth.

We reviewed available English language literature concerning CM and SDB by searching PubMed, using the terms “Chiari, SDB, OSA, and CSA.” This yielded 79 articles between January 1982 and October 2013, with 20 articles (25.3%) describing 100 patients with CM and prominent or presenting sleep-disordered features containing sufficient information for detailed review (for details, see Table 4). In 36% of patients, there was evidence for BI, while 45% had no evidence for BI, and 19% had insufficient information about possible association of BI.

**Table 4:** **Sleep-disordered breathing and Chiari 1 malformation**.

Reference	No. patients (gender)	Age, mean (±SD, and/or range)	Associated BI	Apnea type	Apnea as presenting manifestation?	Surgery	Outcome
Aarts et al. (4)	1 (F)	4	Yes	CSA	Yes	Yes	Improved, but persistent severe CSA
Abel et al. (23)	1 (F)	7	Yes	CSA	Yes	Yes	Improved, but required NIPPV during sleep
Bachetti et al. (24)	1 (F)	20	No	Hypoventilation	No	No	Long-term ventilation
Botelho et al. (9)	23 (16 F/7 M)	43 ± 9.4	Yes (in 9)	Predom. OSA, CSA	Unknown	No	Unknown
Botelho et al. (7)	17 (8M,9F)	43 ± 17 (38–49)	Yes (in 6)	10+ OSA, 7 CSA	Unknown	Yes	Details unknown; entire group, mean AHI improved, mean CAI resolved
Brown et al. (25)	1 (F)	12	Unknown	CSA	Yes	Yes	Resolved
Dauvilliers et al. (11)	28 (13 M, 15F)	Mean children (*n* = 15) = 16, mean adults (*n* = 25) = 37	Yes (in 9)	20 OSA, 8 CSA, 2 hypoventilation	Yes	No	Unknown
Doherty et al. (8)	1 (M)	62	Yes	OSA	Yes	Yes	Improved, but recurrent OSA
Gagnadoux et al. (12)	12 (7M, 5F)	39 (18–62)	Unknown	2 CSA, 5 OSA, 5 Mixed OSA/CSA	Unknown	Yes	Resolved in 2, improved in 2, unchanged in 2, unknown in 6
Gladding and Whyte (26)	1 (M)	22	Yes	OSA, hypoventilation	Yes	Yes	Unchanged; NIPPV for severe OSA
Gosalakkal (27)	1 (F)	13	Yes	CSA	Yes	Yes	Improved
Gupta et al. (28)	1 (F)	72	Yes	CSA	Yes	Yes	Resolved
Hershberger and Chidekel (29)	1 (F)	3	Yes	CSA	Yes	Yes	Improved
Lam and Ryan (30)	1 (M)	39 (M)	No	Mixed OSA/CSA (Comp SAS)	Yes	Yes	Improved
Murray et al. (13)	3 (3F)	3, 9, 13	Yes (in 1), other 2 unknown	CSA	Yes	Yes	Resolved
Rabec et al. (31)	2 (2M)	14, 39	Yes (in 14 years), unknown in other	1 CSA, 1 hypoventilation	Yes	No	CPAP in one; mechanical ventilation in one
Spence et al. (15)	2 (1M, 1F)	7 (F), 15 (M)	Yes (in 15 years), unknown in other	CSA	Yes	Yes	Resolved
Tran and Hukins (32)	1 (F)	19	Yes	CSA, OSA	Yes	Yes	Resolved
Tsara et al. (33)	1 (M)	32	No	OSA, hypoventilation	Yes	Yes	Unchanged, continued hypoventilation, required NIPPV
Van den Broek (34)	1 (M)	4 months	Yes	CSA	Yes	Yes	Resolved

Seventy-eight patients with CM had sufficient information presented concerning the range of SDB manifestations. Forty-five (58%) had OSA alone, 22 (28%) had CSA alone, 8 (10%) had mixed OSA/CSA, and 6 (8%) had hypoventilation. Forty-eight (62%) CM patients presented with SDB and, of these, 22 (46%) were males. Twenty-four (50%) had OSA, 20 (42%) had CSA, 4 (8%) had mixed OSA/CSA, and 5 (10.4%) had hypoventilation. We speculate that CSA may develop in those patients in whom brainstem compression may result in excessive central chemoreflex sensitivity with consequent hypocapnic CSA.

Surgical decompression was performed in 46 patients, and 28 had sufficient data concerning individual patient outcomes. Seventeen (61%) had improved disordered breathing, 6 (21%) resolved, and 5 (18%) had persisting apnea or hypoventilation requiring positive airway pressure therapy. One surgical series of 17 operated patients provided only pooled group outcomes for polysomnographic measures but demonstrated greater improvements in central than obstructive apnea indices (7).

## Concluding Remarks

In conclusion, our case of a young woman with severe CSA in pregnancy caused by CM with associated BI demonstrated improvement of CSA by ASV therapy, suggesting that ASV may be useful in cases of CSA refractory to nasal CPAP therapy. Anesthesia-associated complications of CM must be anticipated and explained to patients before surgery. Near complete resolution of CSA followed surgical decompression, and lack of recurrence during second pregnancy suggested CM as the etiology for CSA in our case. In our patient’s case, the diagnosis of CM was delayed initially given her pregnancy (which precluded definitive MRI imaging), and further delayed by her reassuringly normal neurological examination and lack of other apparent symptoms of brainstem compression. Fortunately, in our patient’s case, the diagnostic delay did not impact her ultimately favorable outcome following decompressive surgery. However, neuroimaging with brain MRI should be considered in patients with severe CSA to exclude central nervous system lesions when no alternative etiology is evident, even when neurological examination is normal. A review of available literature concerning CM and associated SDB manifestations demonstrated that OSA is the most common type. The spectrum of disordered breathing also includes CSA, mixed OSA/CSA, and hypoventilation; and surgical decompression either improves or resolves disordered breathing in nearly 80% of operated patients. However, future prospective treatment trials are necessary to determine optimal PAP-therapy management for CSA associated with CM; and larger prospective surgical series analyzing SDB outcomes are needed.

## Author Contributions

Erik K. St. Louis – drafting and critical revision of the manuscript, literature review, study design, analysis. Praveen Jinnur – critical revision of the manuscript. Stuart J. McCarter – quantitative REM sleep muscle tone analysis. Ethan Duwell – quantitative REM sleep muscle tone analysis. Eduardo E. Benarroch – critical revision of the manuscript. Kejal Kantarci – critical revision of the manuscript. Mark A. Pichelman – critical revision of the manuscript. Michael H. Silber – critical revision of the manuscript. Bradley F. Boeve – critical revision of the manuscript. Eric J. Olson – critical revision of the manuscript. Timothy I. Morgenthaler – critical revision of the manuscript. Virend K. Somers – critical revision of the manuscript.

## Conflict of Interest Statement

Erik K. St. Louis reports grants from Mayo Clinic CTSA, during the conduct of the study; other from Inspire, Inc., outside the submitted work. Bradley F. Boeve reports that he is an investigator in clinical trials sponsored by Cephalon, Inc., Allon Pharmaceuticals, and GE Healthcare. He receives royalties from the publication of a book entitled Behavioral Neurology of Dementia (CambridgeMedicine, 2009). He has received honoraria from the American Academy of Neurology. He serves on the board of the Tau Consortium. He receives research support from the National Institute on Aging [P50 AG16574 (Co- Investigator), U01 AG06786 (Co-Investigator), RO1 AG32306 (Co-Investigator)] and the Mangurian Foundation. Praveen Jinnur, Stuart J. McCarter, Ethan Duwell, Eduardo E. Benarroch, Kejal Kantarci, Mark A. Pichelman, Michael H. Silber, Eric J. Olson, Timothy I. Morgenthaler and Virend K. Somers have nothing to disclose.
